# Periodontal structures in horses with pituitary pars intermedia dysfunction: A histological evaluation

**DOI:** 10.3389/fvets.2023.1114445

**Published:** 2023-01-17

**Authors:** Anne Maria Zapf, Kerstin Fey, Kathrin Büttner, Manuela Gröf, Carsten Staszyk

**Affiliations:** ^1^Equine Clinic, Internal Medicine, Faculty of Veterinary Medicine, Justus-Liebig-University, Giessen, Germany; ^2^Unit for Biomathematics and Data Processing, Faculty of Veterinary Medicine, Justus-Liebig-University, Giessen, Germany; ^3^Faculty of Veterinary Medicine, Institute of Veterinary Pathology, Justus-Liebig-University, Giessen, Germany; ^4^Faculty of Veterinary Medicine, Institute of Veterinary-Anatomy, Histology, and Embryology, Justus-Liebig-University, Giessen, Germany

**Keywords:** pituitary pars intermedia dysfunction (PPID), periodontal disease (PD), diastema, leukocytic infiltration, histology, equine teeth

## Abstract

**Introduction:**

Pituitary pars intermedia dysfunction (PPID) and dental disorders are of major concern in horses older than 15 years. Although PPID in geriatric horses and dental disorders in all age groups are well described, a connection between this endocrine disease and pathological changes in equine dental structures has not yet been investigated. In humans, periodontitis is considered to be a complication of systemic diseases like diabetes mellitus type 2, obesity and various conditions leading to an impaired immune response. In PPID, cross links to insulin and immune dysregulations are proven. The aim of this study was to compare histological findings of the gingiva and the sub gingival periodontal ligament of PPID affected horses with control horses.

**Methods:**

In a case-control morphometric descriptive study, 145 dental locations of 10 PPID affected horses (27.3 ± 2.06 years) were compared with 147 dental locations of 10 controls (21.4 ± 4.12 years). Histological parameters were leukocyte infiltration, keratinization of gingival epithelium, blood vessel supply of the periodontium and structure of cementum.

**Results:**

The distribution and localization of gingival leukocyte infiltrations (LI) in PPID affected horses was more often multifocal to coalescing (*p* = 0.002) and reached into deeper parts of the periodontium, sometimes down to the sub gingival periodontal ligament (PDL). Aged animals of both groups showed higher prevalence (PPID: OR 1.66; controls: OR 1.15) for severe leukocyte infiltration in the PDL. PPID was not significantly associated with increased LI. The cementum bordering the soft tissue in interdental locations showed four times more irregularities in PPID affected horses than in controls which predisposes for interdental food impaction and periodontal diseases.

**Discussion:**

In summary, multifocal to coalescing leukocytes and irregular cementum are seen more often in PPID than in controls - however our findings mainly reflect an association of older age with periodontal disease.

## Introduction

In human and canine dentistry, precise descriptions of morphological and histological features of the gingiva exist (1–7). In contrast, the gingiva of the horse is addressed only in a few studies (8–10). The gingiva, along with the cementum, periodontal ligament and alveolar bone, forms the structural and functional group of tissue called the periodontium. The epithelium of the healthy gingiva is connected with the alveolar mucosa at the mucogingival junction (MGJ). The gingiva itself can be divided into an attached and a free part. The attached gingiva is firmly bound to the periosteum of the alveolus by connective tissue. The free gingiva lies unattached next to the tooth, forming a collar (5). The space between tooth and free gingiva is named the gingival sulcus (*Sulcus gingivalis*) and the epithelium forming the inner wall of the gingival sulcus is called the sulcular epithelium (11). At the bottom of the gingival sulcus, the so-called junctional epithelium attaches to the hard substance of the tooth. In contrast to brachydont species, where the junctional epithelium attaches to enamel, in hypsodont species like horses it attaches to the dental cementum (11). An intact junctional epithelium forms a permeable barrier with important immunological tasks, protecting the deeper tissues from the oral microbiotic flora and preventing periodontal diseases. In horses, the primary reasons for the onset of periodontal diseases are physical disorders of tooth growth, eruption and/or wear (9, 12, 13), causing gaps (diastemata) between neighboring cheek teeth and allowing food to become entrapped. Once food is impacted in widened interdental spaces, the integrity of the interdental junctional epithelium is lost and progressive periodontal disease results.

The most likely etiopathogenesis of periodontal disease in human and canine species is primarily bacterial, initiated by the development of plaque (3, 9), leading to gingival inflammation as the key factor for the onset of periodontitis (14). In human dentistry, hormonal changes due to puberty or pregnancy lead to changes in the immune system, which predispose for gingivitis (15–17). Hyperglycemia, like in diabetes mellitus type 1 and 2, hypercortisolism in Cushing's syndrome and immunodeficiencies (e.g. in leukemia, neutropenia, acquired immune deficiency syndrome) are also known to modify the course and severity of periodontitis. This proves the multifactorial nature of this seemingly localized inflammation (15, 17–19). Furthermore, obesity is recognized as a risk factor for the development of periodontitis (20). Impaired immune function due to increased production of proinflammatory cytokines from adipose tissue (adipokines) is proposed as the underlining pathophysiological mechanism (21).

PPID is the most common equine endocrine disease, affecting more than 20% of horses older than 15 years. The loss of dopaminergic neurons leads to loss of control of the melanotropic cells in the pars intermedia of the equine pituitary gland (22, 23). The result is an overproduction of proopiomelanocortin (POMC) (24). POMC is further processed into smaller peptides, e.g., adrenocorticotropin (ACTH), α-melanocyte-stimulating hormone (α-MSH), β-endorphin and corticotropin-like intermediate lobe peptide (CLIP). The different amounts and the variety of POMC-derived peptides are thought to cause the different clinical signs of PPID (23, 25). Although PPID is a specific equine endocrine disease, it is often associated with insulin dysregulation, delayed wound-healing, altered collagen metabolism and increased susceptibility to infections (16, 25, 26), which are known to predispose for periodontal disease in humans and dogs. The question arises, if – besides old age – a systemic disease like PPID may weaken the periodontal apparatus, leading to interdental widening, food entrapment and finally to periodontitis. We hypothesized, that periodontal disease in older equids with PPID is more common than in aged controls. Therefore, the main objective of this study was to describe histomorphological characteristics, including inflammatory signs of the equine gingiva and subgingival periodontium by comparing healthy horses older than 15 years with PPID affected horses.

## Materials and methods

### Inclusion criteria

PPID affected horses (*n* = 10) and non-affected controls (*n* = 10) needed to be 15 years or older. The inclusion criteria for the PPID group were the presence of micro- or macroadenoma in the pars intermedia of the pituitary gland and at least one clinical sign of PPID (e.g., hypertrichosis, muscle atrophy leading to a “sway back” or “pot-belly” appearance, abnormal fat distribution, hyperhidrosis, laminitis) or a history of PPID with at least one of the above mentioned clinical signs. The control group consisted of horses without PPID-associated alterations in the pituitary gland, and without a history or clinical signs of PPID (Table 1). The horses were euthanized due to medical reasons unrelated to this study.

**Table 1:** Data of probands.

**No**.	**Age (years)**	**Sex**	**Breed**	**Cause of euthanasia**	**Pergolid therapy^1^**	**Group**	**Pituitary histology score^2^**
1	16	Gelding	Warmblood	Colic	–	Control	2
2	18	Mare	Haflinger	Colic	–	Control	2
3	18	Mare	Draft	Myositis	–	Control	2
4	19	Mare	Icelandic horse	Colitis	–	Control	1
5	20	Mare	Icelandic horse	Strangles and musculoskeletal problems	–	Control	2
6	21	Gelding	Haflinger	Colic	–	Control	2
7	22	Mare	Friesian	Colic	–	Control	1
8	25	Mare	Warmblood	Colic	–	Control	2
9	26	Gelding	Icelandic horse	Colic	–	Control	2
10	29	Gelding	Pony	Colic	–	Control	2
11	24	Gelding	Warmblood	Neoplasia	–	PPID	4
12	25	Gelding	Warmblood	Colic	+ n.k.	PPID	5
13	25	Mare	Haflinger	Spine fracture	–	PPID	5
14	27	Mare	Warmblood	Neoplasia	1 mg^*^	PPID	5
15	27	Gelding	Warmblood	Dysphagia		PPID	5
16	28	Mare	Warmblood	Colic	2 mg^*^	PPID	4
17	29	Gelding	Warmblood	Colic	–	PPID	5
18	29	Mare	Icelandic horse	Hoof abscesses	–	PPID	4
19	29	Gelding	Pony	Colic	+ n.k.	PPID	5
20	30	Gelding	Pony	Laminitis	1.25 mg^*^	PPID	5

1 Pergolide, a dopamine agonist, commonly administered to manage PPID.

2 According to Miller et al. (27). n.k., not known.

* Per animal and day.

### Dissection and staining

**Figure 1:**
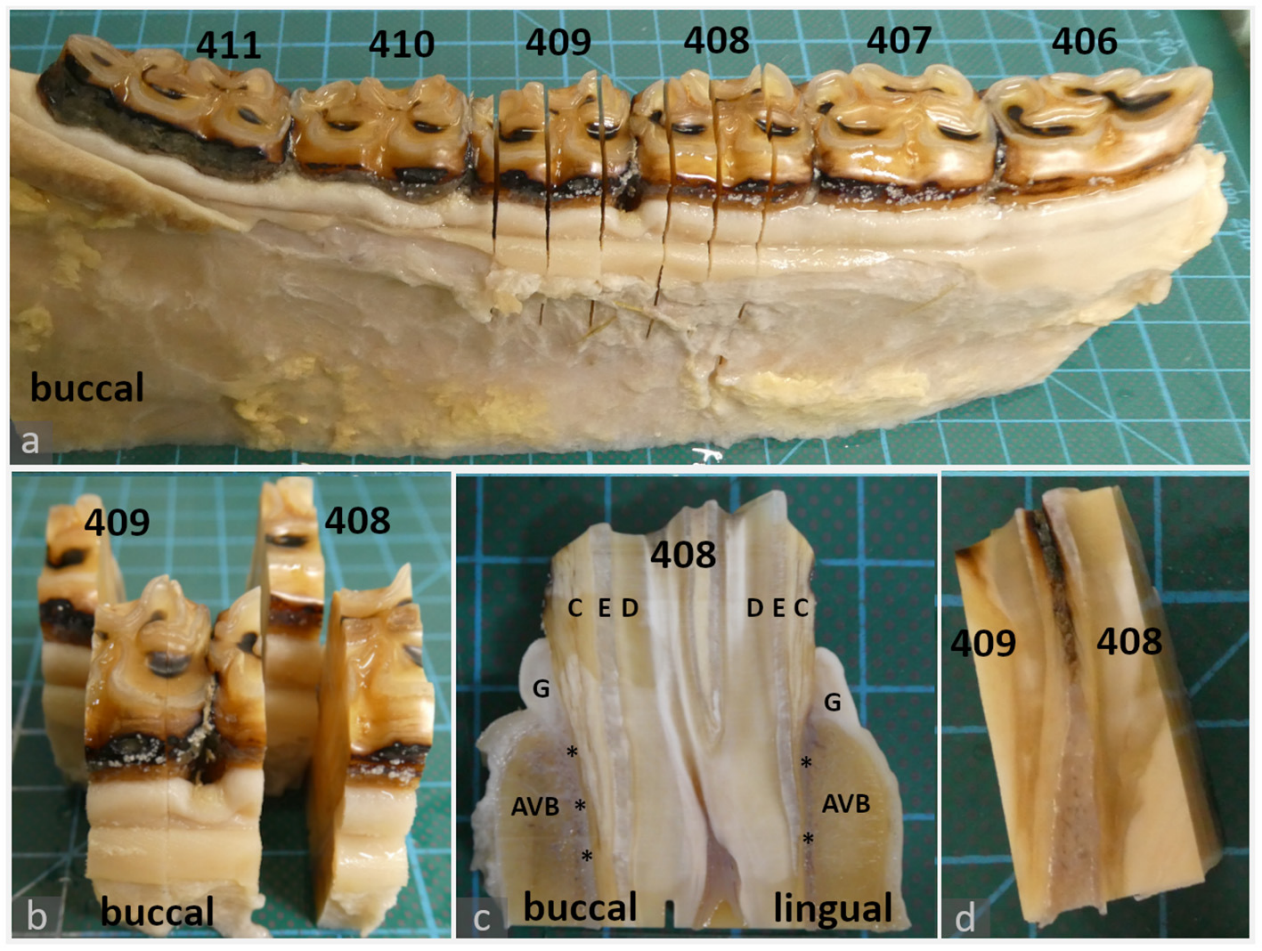
Histologic sampling, examples shown for teeth 408 and 409 **(a–c)**: 20-year-old Icelandic mare (control) **(d)**: 29-year-old Warmblood mare (PPID). **(a, b)**: Dental gingiva samples were collected by transverse section of teeth 408 and 409 in mesial and distal positions of the tooth. The mesial position was processed and evaluated as described in material and methods. The distal position (background) was archived. **(c)**: Sections were further divided to obtain separate specimens from the lingual and buccal aspect. Each specimen contained the gingiva (G), subgingival parts of the alveolar bone (AVB), subgingival periodontal ligament (asterisks) and dental substances: cement (C), enamel (E), dentin (D). **(d)**: Interdental gingiva samples were collected by sagittal section through teeth 408 and 409.

**Figure 2:**
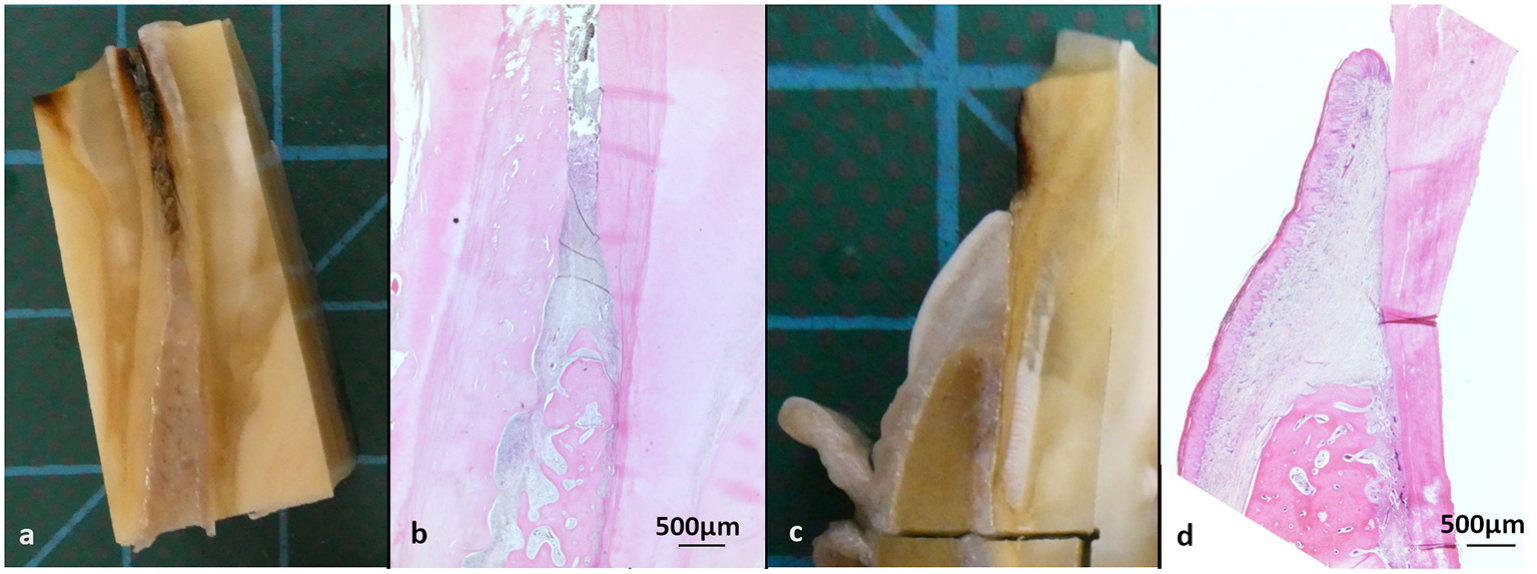
Macroscopic and histological overview, stained with hematoxylin-eosin (HE). **(a, b)** Interdental ginigva sample between tooth 408 and 409 of a 29-year-old Icelandic mare (PPID). **(c, d)** Dental gingiva sample, cheek teeth 409 lingual aspect of a 19-year-old Icelandic mare (control).

The jaws were dissected with a band saw (type K440H, Kolbe Foodtec, Elchingen, Germany) to obtain the upper and lower incisor arcades as well as the four cheek teeth quadrants. The specimens were cleaned of loose food and debris with water and were subsequently fixed in 10% neutral buffered formalin. Macroscopic findings such as periodontal pocketing, diastema formation, food impaction and gingival texture changes are published elsewhere (28). The specimens were further sectioned to obtain histological samples from preassigned locations (Table 2, Figures 1, 2) with a diamond bladed band saw (Proxxon Type MBS 240/E No 27 172, Föhren, Germany). The samples were processed as described by Steinfort et al. (8). Specimens were decalcified in buffered ethylene diamine tetra-acetate (EDTA, pH 8.0) for 6–8 weeks at room temperature. Afterwards, the specimens were embedded in para?n wax, sectioned and stained with hematoxylin-eosin (HE). Samples were assessed via light microscopy (Leica DM750, Leica DM2500 and Leica ICC50 HD, Wetzlar, Germany). With slight modifications, the system of Steinfort et al. (8) was used to evaluate the following parameters in the dental and interdental gingiva and the subgingival periodontal tissue:

**Table 2:** Locations and numbers of histologic specimens.

**No**.	**Teeth**	**Direction**	**Localization**	***n* (PPID)**	***n* (Control)**
1	101/102	Interdental	Labial	8	9
2	102	Mesial	Labial	10	10
3	102	Mesial	Palatal	10	10
4	108	Mesial	Buccal	10	10
5	108	Mesial	Palatal	10	10
6	108/109	Interdental	Buccal	7	8
7	109	Mesial	Buccal	10	10
8	109	Mesial	Palatal	10	10
9	401/402	Interdental	Labial	8	7
10	402	Mesial	Labial	10	9
11	402	Mesial	Lingual	10	9
12	408	Mesial	Buccal	9	10
13	408	Mesial	Lingual	9	10
14	408/409	Interdental	Buccal	6	7
15	409	Mesial	Buccal	9	9
16	409	Mesial	Lingual	9	9

### Oral gingival epithelium, sulcular epithelium (SE), junctional epithelium (JE)

#### Keratinization type and presence of rete pegs

Epithelia were evaluated for the type of keratinization: non-keratinized, orthokeratinized (no nuclei in stratum corneum) and parakeratinized (cells with pyknotic nuclei in stratum corneum). In addition, the presence/absence of rete pegs was recorded.

#### Leukocyte infiltrations

SE and JE were each evaluated for the presence of locally or diffusely distributed leukocytic infiltration (LI) and leukocyte cell type, using 400x total-magnification.

The degree of LI was scored using 200x total-magnification within the complete SE and JE. For statistical reasons, the scores were summarized in the binominal categories of either “low” or “high”:

Low LI = 0: no leukocytic infiltration; 1: 1–2 leukocytes (mild);High LI = 2: 3–10 leukocytes (moderate); 3: > 10 leukocytes (severe).

#### Attachment area of the JE

The attached area of the JE on the cementum surface was scored as 0: no attachment; 1: attachment present with 1-5 cell layers; 2: attachment present with > 5 cell layers. For statistical reasons the scores 1 and 2 were combined to create the binominal categories of either “no attachment” or “attachment present”.

### Lamina propria (LP) and subgingival periodontal ligament (PDL)

#### Leukocytic infiltration

The distribution of leukocytes in the LP and PDL was recorded concerning localization (e.g., close to the gingival sulcus, the cementum, the oral gingival epithelium or the alveolar bone) and categorized as not present, multifocal or multifocal to coalescing.

The leukocyte cell types were evaluated in 400x total magnification.

The degree of LI was scored using 100x total-magnification within the complete LP. The following score was applied:

Low LI = 1: 0–50 leukocytes (mild);

High LI = 2: 51–100 leukocytes (moderate); 3: > 100 leukocytes (severe).

Eosinophilic infiltration was recorded separately as present or not present.

#### Blood vessels

The distribution and localization of blood vessels in the lamina propria and the PDL was recorded and grouped either as homogenous (physiologic) or inhomogeneous, their quantity was scored using one optic field in 100x total-magnification:

Low numbers of blood vessels = 0–5 blood vessels;

High numbers of blood vessels = ≥ 6 blood vessels.

#### Collagen fiber arrangement

The collagen fiber arrangement was evaluated in the LP and PDL as regular or irregular (fibers running in criss-cross manner with irregular distances between connective tissue cells and matrix) in HE stain.

### Cementum

#### Cemental structure

Higher mineralized cementum from “rest periods” in cementum formation was visible as parallel incremental lines as described by Fiorellini et al. (29). Evaluation of the alignment of incremental lines was recorded as regular or irregular.

#### Plaque and/or caries

The presence and localization of plaque (bluish deposition on cemental surface) and/or caries [brownish discoloration of the bluish plaque matrix and loss of calcified tissue forming flake or flask-like lesions, were defined as peripheral caries (30)] was recorded as present or not present, and classified as supragingival (above the sulcus gingivalis) or gingival (within the gingival sulcus).

### Statistical analysis

The dataset was analyzed with the statistical software SAS^®^ 9.4 (SAS Institute Inc., 2008). A *t*-test was calculated to detect the age difference between the groups. In all epithelia, keratinization was evaluated as present or not present. The degrees of leukocytic infiltration of the sulcular epithelium and the lamina propria were combined for statistical analysis (= degree of leukocytic infiltration of the gingiva: LIG). LIG was differentiated in “low” (0–2 leukocytes in SE, 0–50 leukocytes in LP) or “high” (≥ 3 leukocytes in SE, ≥ 51 leukocytes in LP). The junctional epithelium was evaluated separately because of its specialized tasks in the periodontium. Plaque was almost ubiquitous. Therefore, just localization of plaque was compared statistically between groups. Caries was categorized as present or absent.

All dependent variables, except the distribution of leukocytes in the PDL in dental and interdental locations, were analyzed using the GLIMMIX procedure with a binomial distribution (link-functio*n* = logit). For the distribution of leukocytes in the PDL in dental and interdental locations a multinomial model was created (link-functio*n* = cumlogit). The models included age as a covariable and group as a fixed effect. The interaction between age and group was also tested. If there was no significant interaction between age and group, the interaction was removed from the model. All model results were calculated for a mean age of 24.4 years. Due to rare occurence in some of the tested parameters (dental gingiva locations: oral gingival and junctional epithelium keratinization type, oral gingival and sulcular epithelium presence of rete pegs, PDL distribution of blood vessels; interdental gingiva locations: oral gingival epithelium presence of rete pegs, LP and PDL collagen fiber arrangement, PDL distribution of blood vessels and eosinophil infiltration) fitting of the model was not possible. In these cases, a Chi-Square test or Fisher exact tests were used to test the dependency of the group and the respective parameter. Age could not be considered in these parameters.

All statistical analyses were carried out separately for dental gingiva and interdental gingiva samples. Data is presented as mean ± SD if not mentioned otherwise. In all cases, a significance level of *p* ≤ 0.05 was used.

## Results

### Proband characteristics

The PPID group (*n* = 10) consisted of four mares and six geldings, aged from 24 to 30 years (27.3 ± 2.06), including six Warmbloods, two Ponies, one Icelandic Horse and one Haflinger. Treatment with pergolid was reported in five horses of the PPID group. The control group (*n* = 10) consisted of six mares and four geldings, aged from 16 to 29 years (21.4 ± 4.12), including three Icelandic Horses, two Warmbloods, two Haflingers, one Pony, one Friesian and one Draft Horse. Horses in the PPID-affected group were significantly older than in the control group (*p* = 0.0007).

### Histological findings in the dental gingiva

Table 3 summarizes the histological findings in the dental and interdental gingiva.

**Table 3:** Characteristics of the histology of the equine periodontium in PPID and control horses.

	**Dental gingiva**			**Interdental gingiva**		
	**PPID**	**Controls**	**p-value** **age**	**PPID**	**Controls**	**p-value group**
**Junctional epithelium**						
*Distribution of leukocytic infiltration:*			0.66 0.526			–
No LI present	18.2% (18/99)	15.8% (18/114)		–	–	
Local to diffuse LI present	81.8% (81/99)	84.2% (96/114)		–	–	
*Amount of leukocytic infiltration:*			0.054 0.353			–
Low (0-2 leukocytes)	34.3% (34/99)	38.6% (44/114)		–	–	
High (≥ 3 leucocytes)	65.7% (65/99)	61.4% (70/114)		–	–	
**Gingiva (lamina propria and sulcular epithelium)**						
*Distribution of leukocytic infiltration:*			0.397 **0.002**			0.95 0.13
Multifocal	69.3% (79/114)	91.4% (106/116)		72.4% (21/29)	93.5% (29/31)	
Multifocal to coalescing	30.7% (35/114)	8.6% (10/116)		27.6% (8/29)	6.5% (2/31)	
*Amount of leukocytic infiltration:*			0.497 0.459			0.52 0.45
Low (0-2 LI in SE, 0-50 LI in LP)	12.3% (14/114)	20.7% (24/116)		27.6% (8/29)	22.6% (7/31)	
High (≥ 3 -LI in SE, ≥ 51 LI in LP)	87.7% (100/114)	79.3% (92/116)		72.4% (21/29)	77.4% (24/31)	
Eosinophilic infiltration	40.4% (46/114)	56.0% (65/116)	0.10 0.60	27.6% (8/29)	41.9% (13/31)	0.295 0.125
***Ligamentum periodontale (PDL)***						
*Distribution of leukocytic infiltration:*			**0.049** 0.217			0.666 0.865
No leukocyte infiltration	13.3% (15/113)	33.6% (38/113)		11.1% (2/18)	26.1% (6/23)	
Multifocal	65.5% (74/113)	54.0% (61/113)		50.0% (9/18)	34.8% (8/23)	
Multifocal to coalescing	21.2% (24/113)	12.4% (14/113)		38.9% (7/18)	39.1% (9/23)	
*Amount of leukocytic infiltration:*			**< 0.0001** 0.11			**0.028** 0.91
Low (0-50 leukocytes)	56.6% (64/113)	69.9% (79/113)		55.6% (10/18)	82.6% (19/23)	
High (≥51 leukocytes)	43.4% (49/113)	30.1% (34/113)		44.4% (8/10)	17.4% (4/23)	
Eosinophilic infiltration	10.6% (12/113)	2.7% (3/113)	0.278 0.312	22.2% (4/18)	0.0% (0/23)	n.a. **0.03**^**+**^
**Cementum (surface)**						
Irregular cemental structure	47.8% (55/115)	16.4% (19/116)	age^*^group **0.005**	51.7% (15/29)	12.9% (4/31)	0.795 **0.024**
Presence of plaque within sulcus gingivalis	42.1% (48/114)	31.0% (36/116)	0.78 0.31	79.3% (23/29)	83.9% (26/31)	0.39 0.37
Presence of caries	29.8% (34/114)	39.7% (46/116)	age^*^group **0.007**	31.0% (9/29)	51.6% (16/31)	0.72 0.15

Displayed are the percentages of observations, absolute numbers are given in brackets. P-values are given after computational adjustment of the data to the mean age of 24.4 years in both groups. +: logistic regression not possible due to small numbers of findings in the categories (p-value of exact fisher test). Significant p-values are presented in bold letters. The interaction age^*^group is significant only if displayed. The p-values of age and group are not displayed in this case, as they cannot be interpreted separately from each other. n.a., not accessible due to too small numbers.

#### Oral gingival epithelium

A parakeratinized stratified squamous epithelium was found in 99.1% (115/116) of the PPID and in 79.1% (91/115) of the control specimens. Only one PPID sample (0.9%) featured a non-keratinized stratified squamous epithelium (108 palatinal), whereas in controls, 20.9% of the specimens showed an orthokeratinized stratified squamous epithelium. Rete pegs were visible in all specimens and no blood vessels or leukocyte infiltrates were found in the oral gingival epithelium in either group. No significant differences between the groups were found.

#### Sulcular epithelium

In the PPID group, 50.9% of the specimens showed a keratinized stratified squamous epithelium, while in the control group 44.1% were keratinized (*p* = 0.3). None of the specimens showed orthokeratinization. In both groups, the coronal aspect of the SE was usually keratinized, and the apical aspect showed a non-keratinized stratified squamous epithelium. Rete pegs were visible in all PPID samples and in 97.4% (112/115) of the control specimens.

In both groups, approximately 15% of specimens showed no leukocytes within their SE. For statistical analysis, the degrees of LI in SE and LP were combined to leukocytic infiltration of the gingiva (LIG) and are displayed in the LP section. In both groups, the cell type of the LI in the SE consisted predominantly of lymphocytes, followed by neutrophils. Plasma cells were present only occasionally.

#### Junctional epithelium

**Figure 3:**
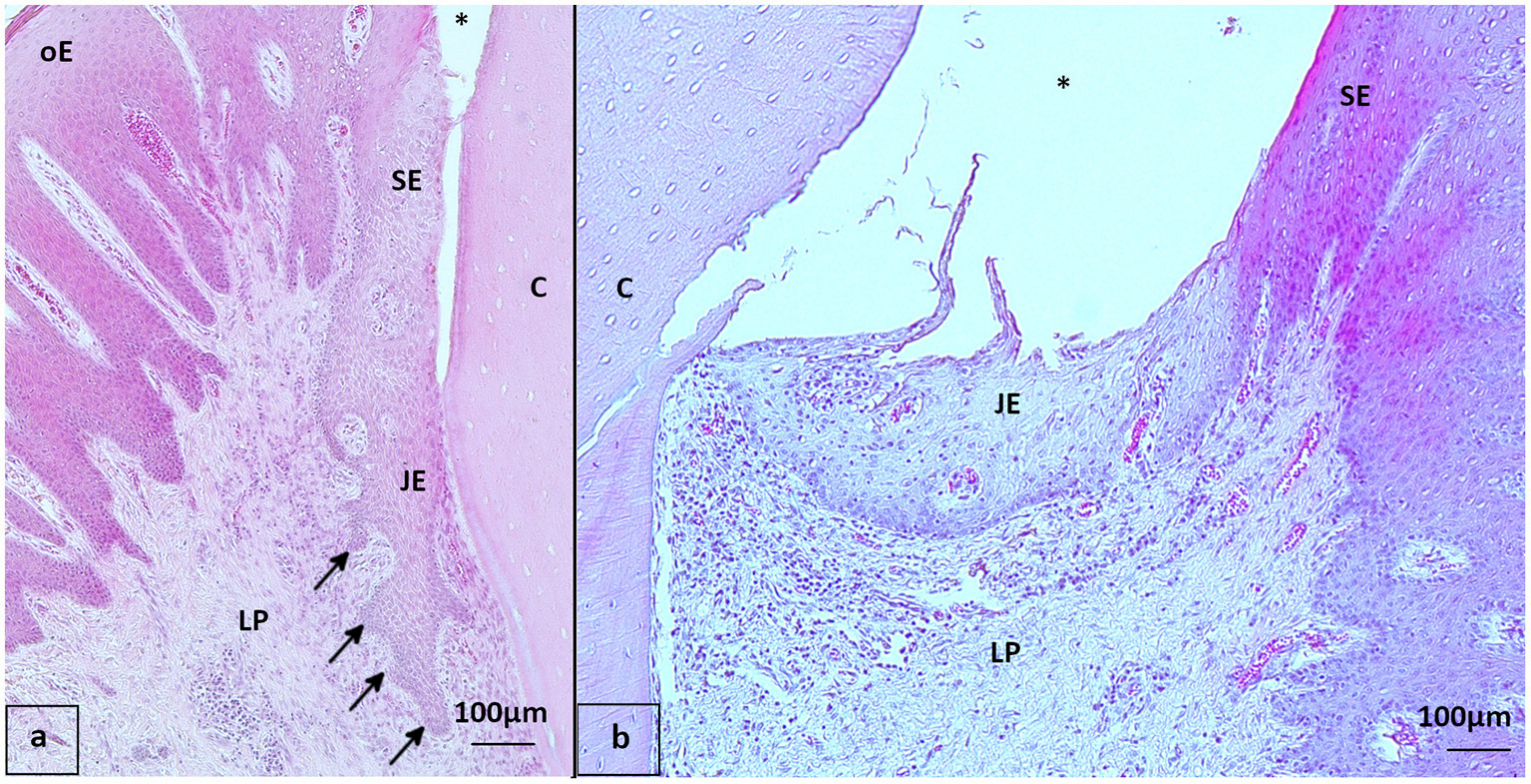
Gingival sulcus and junctional epithelium with and without rete pegs, staining hematoxylin-eosin (HE) **(a, b)** oE, Oral epithelium; SE, Sulcular epithelium; JE, Junctional epithelium; LP, Lamina propria; C, Cementum; ^*^Sulcus gingivalis **(a)** 22-year-old Friesian mare, control, 108 buccal aspect: Rete pegs (arrows) in JE present **(b)** 25-year-old Warmblood gelding, PPID, 109 buccal aspect: Rete pegs in JE not visible, notice leukocyte infiltration beneath and inside the JE.

In both groups, the JE was classified as a non-keratinized stratified squamous epithelium. After controlling for age, rete pegs were present in 5.2% (5/97) of PPID specimens and 12.4% (14/113) of controls (Figure 3). Neither group nor age showed a significant influence on the presence of rete pegs. LI were present in 81.8% (81/99) of PPID samples and in 84.2% (96/114) of controls. Neither group (*p* = 0.35) nor age (*p* = 0.054) showed an influence on the degree of leukocytic infiltration in the JE. Like in the SE, the predominant cell types were lymphocytes, followed by neutrophils and plasma cells. An intact attachment of the JE to the tooth was not more often seen in the control group (96.5%) than in the PPID group (92.8%; *p* = 0.64).

#### Lamina propria

**Figure 4:**
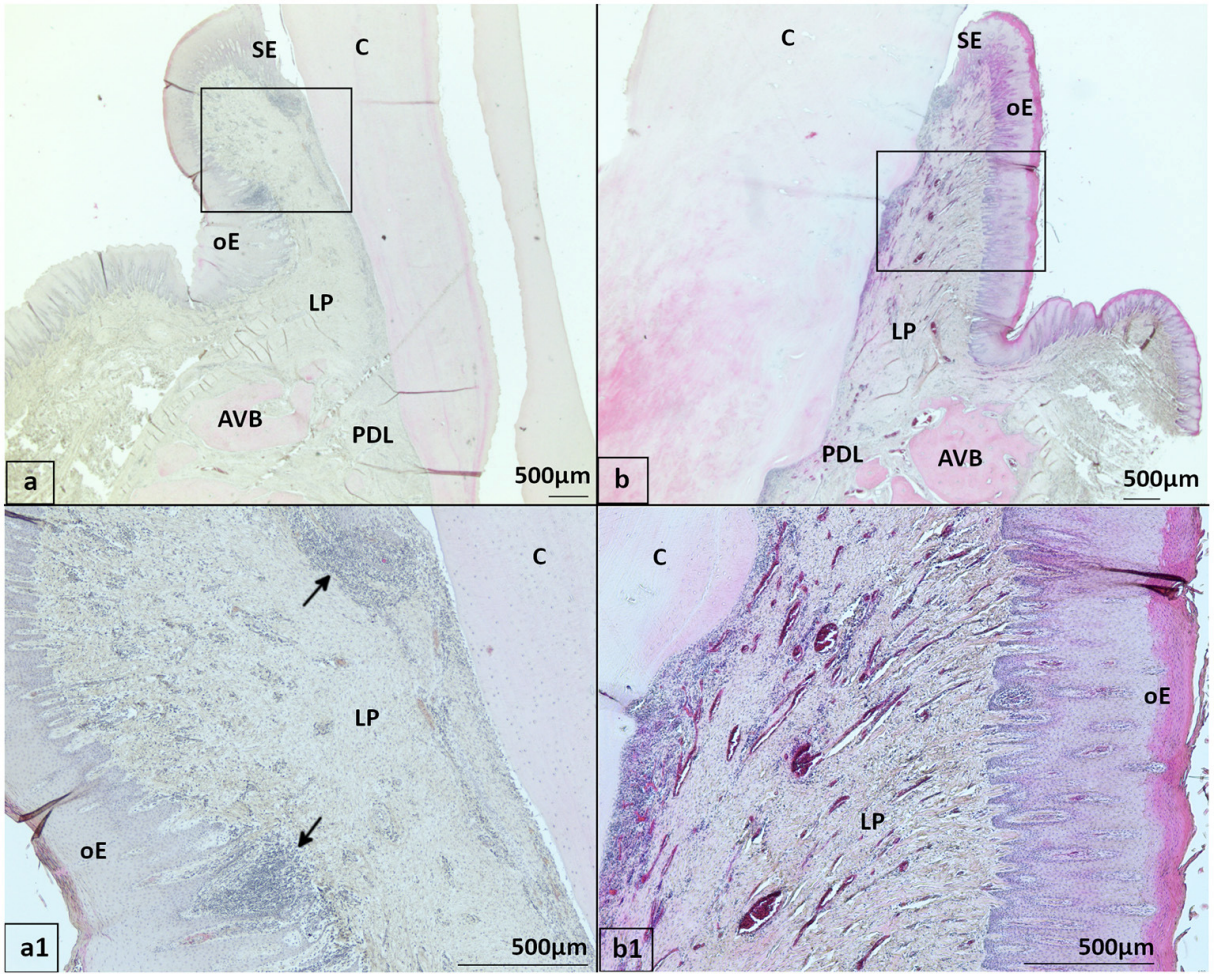
Distribution of leukocyte infiltrations (LI) in gingiva, HE stained **(a, a1)** 20-year-old Icelandic control mare, 409 buccal aspect with multifocal LI (arrows) in gingiva close to the gingival sulcus and oral epithelium. **(b, b1)** 29-year-old Warmblood gelding with PPID, 408 buccal aspect with multifocal to coalescing LI in the gingiva. oE, Oral epithelium; SE, Sulcular epithelium; LP, Lamina propria; PDL, Periodontal ligament; AVB, Alveolar bone; C, Cementum.

The LP contained homogenously distributed blood vessels and a subepithelial capillary plexus beneath the JE and SE in every specimen. The number of blood vessels in the LP did not differ between the groups. Perivascular leukocyte infiltration was seen in all specimens of both groups in the gingiva. The PPID group showed more often (87.7%) a high degree of leukocytic infiltration of the gingiva (LIG) than the controls (79.3%), but there were no statistically significant differences, even if the adjustment for a mean age of 24.4 years was applied. However, the distribution of LI in the lamina propria showed a significant difference between both groups (*p* = 0.002). In the PPID group, 65.4% of the specimens showed multifocal LI and 34.6% multifocal to coalescing LI. Controls showed multifocal LI in 92.8% and multifocal to coalescing LI in only 7.2% (Figure 4). When comparing the groups at the mean age of 24.4 years, PPID affected animals showed a higher chance (OR: 6.83) for a multifocal to coalescing LI distribution in the LP than controls. The leukocyte cell populations consisted predominantly of lymphocytes and plasma cells. The third most common cell population in both groups in the LP were eosinophils. Eosinophils were observed in both groups as focal or multifocal infiltrations, lying close to the gingival sulcus or the cementum. Neither the group nor age influenced significantly the presence of eosinophils in the LP. The PPID group showed slightly fewer eosinophilic infiltrations (45.8%), than the controls (50.7%). Macrophages and neutrophils were never seen as the dominant cell population but were present, especially if there were macroscopically noted periodontal pockets close to the histological sampling location.

#### Subgingival periodontal ligament

**Figure 5:**
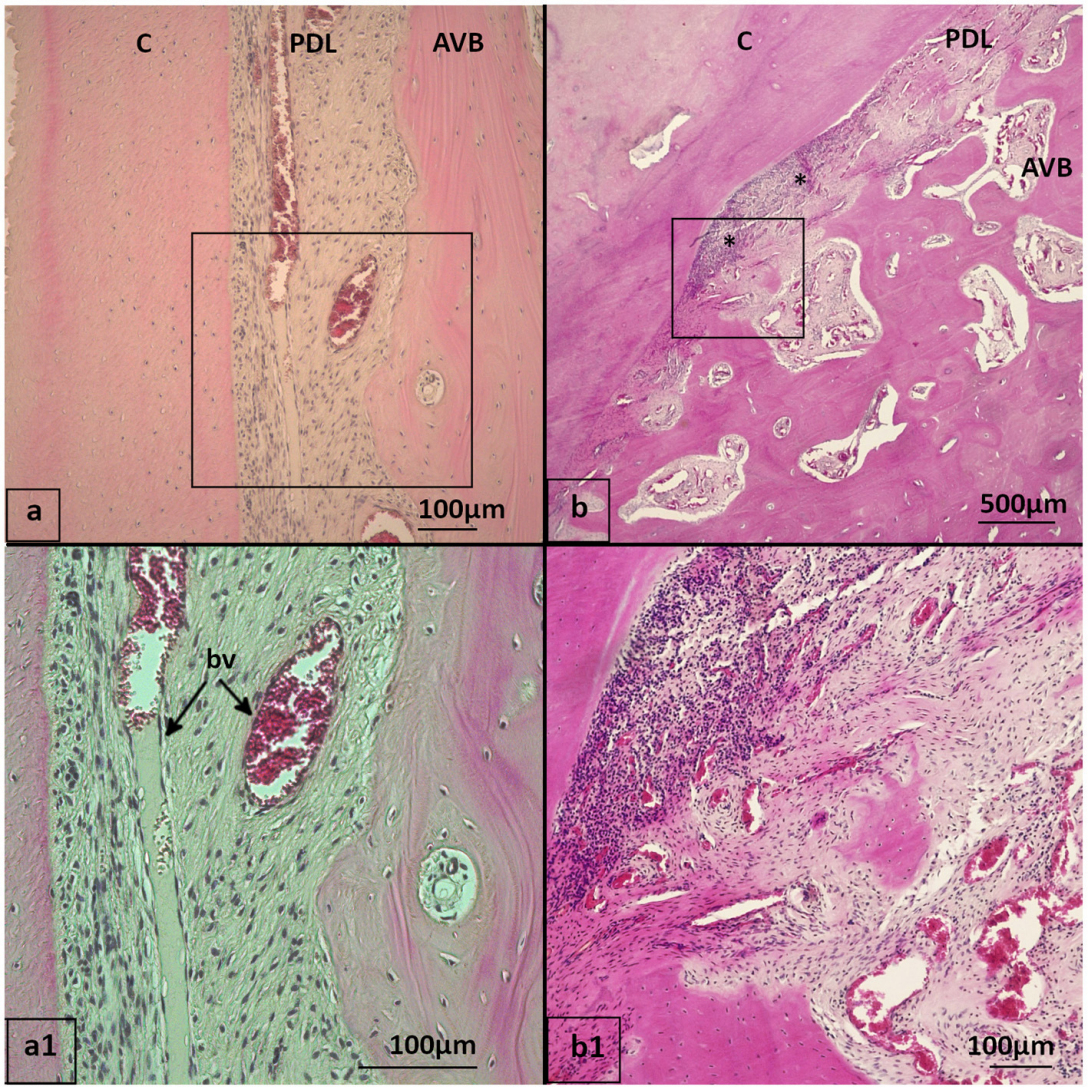
Leukocyte infiltrations (LI) in PDL, HE stained **(a, a1)** 21-year-old Haflinger gelding of the control group, 108 buccal aspect with no LI in PDL. The tooth position showed a gingival sulcus depth of < 1 mm and macroscopic gingiva alterations. No leukocyte infiltration within the PDL. Bv, blood vessels in longitudinal direction in the PDL. **(b, b1)** 28-year-old Warmblood mare with PPID, 409 buccal aspect with focal LI (^*^) in the PDL without periodontal pocket close to the observed area. The tooth position showed a gingival sulcus depth of 3 mm and macroscopical alterations of the gingiva. PDL, Periodontal ligament; AVB, Alveolar bone; C, Cementum.

**Figure 6:**
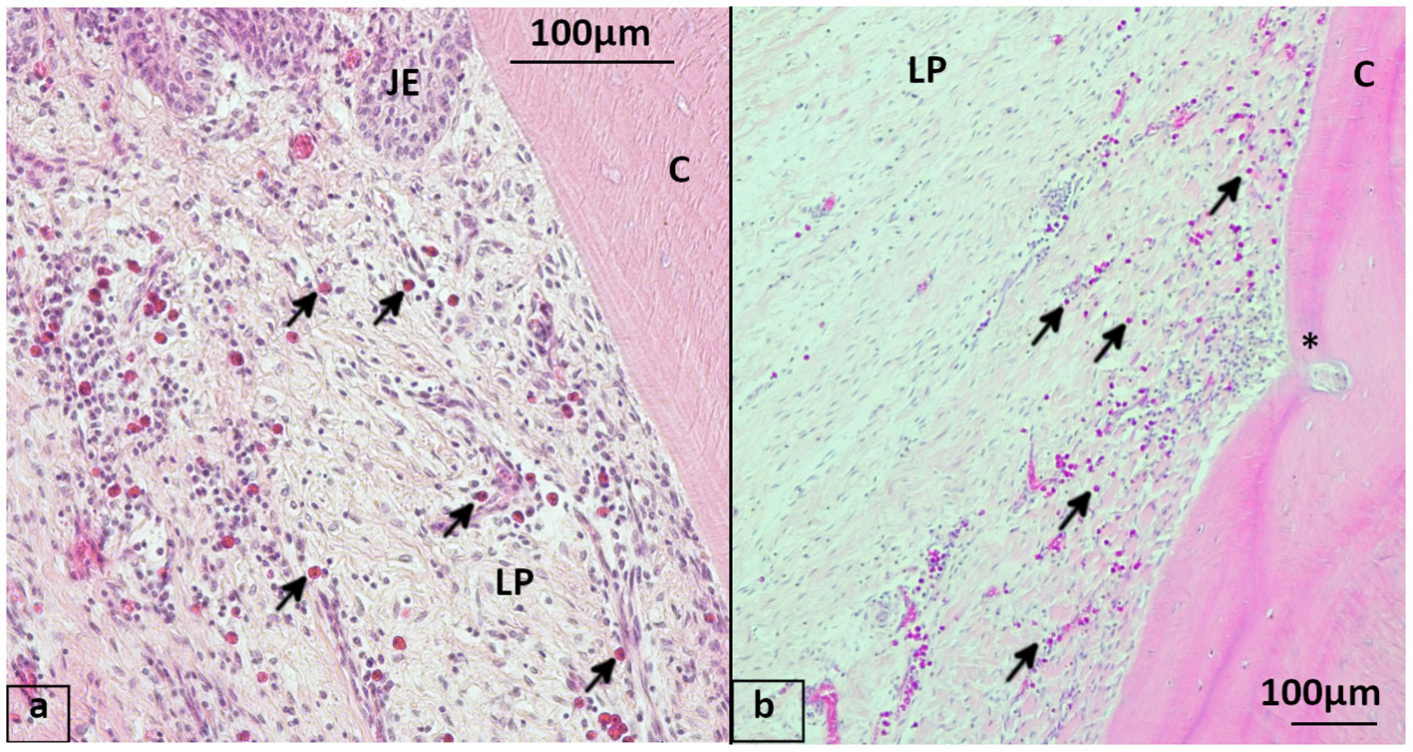
Eosinophils in the gingiva, staining hematoxylin-eosin (HE) **(a)** 19-year-old Icelandic mare from the control group, 409 lingual aspect with mulitfocal perivascular eosinophil infiltration (arrows) of the lamina propria beneath the junctional epithelium. **(b)** 29-year-old Icelandic PPID mare, 408 lingual aspect showing multiple eosinophils infiltrating the lamina propria, blood vessel running into cementum (^*^). JE, Junctional epithelium; LP, Lamina propria; C, Cementum.

Blood vessels in the PDL of both groups were observed in every specimen, lying either close to the alveolar bone or close to the cementum in a homogenous distribution. Most of the specimens (65.8% in PPID and 75.8% in controls) showed high blood vessel numbers with no significant difference. Age but not group, had a major effect (p < 0.0001) on the amount of LI in the PDL. Animals older than 24.4 years had a higher chance (OR: 1.24) for high amounts of LI in the PDL (Figure 5). In the PPID group, 13.3% (15/113) showed no LI in the PDL, 65.5% showed multifocal LI and in 21.2%, a multifocal to coalescing distribution of LI in the PDL was noted. In the control group, 33.6% (38/113) showed no LI, 54% showed a multifocal distribution of LI and 12.4% a multifocal to coalescing LI in the PDL. Even after mathematically controlling for age, no significant effect on the distribution of LI in the PDL between groups was calculated. However, if the age decreases by one year, the chance to show none or multifocal LI in the PDL increases (OR: 1.1; *p* = 0.05). Leukocyte cell populations consisted predominantly of lymphocytes and plasma cells in both groups with some apoptotic lymphocytes. Eosinophils were seen more frequently in the PPID group (10.6%) than in the controls (2.7%), but neither group nor age showed a significant effect on the presence of eosinophils in the PDL (Figure 6). Neutrophils and macrophages were never the dominant cell population, but single such cells were observed in individual samples from both groups. There was no significant difference between the two groups concerning the presence of an irregular collagen fiber network (PPID 5.6%; controls 2.6%).

#### Peripheral (dental gingiva) cementum

**Figure 7:**
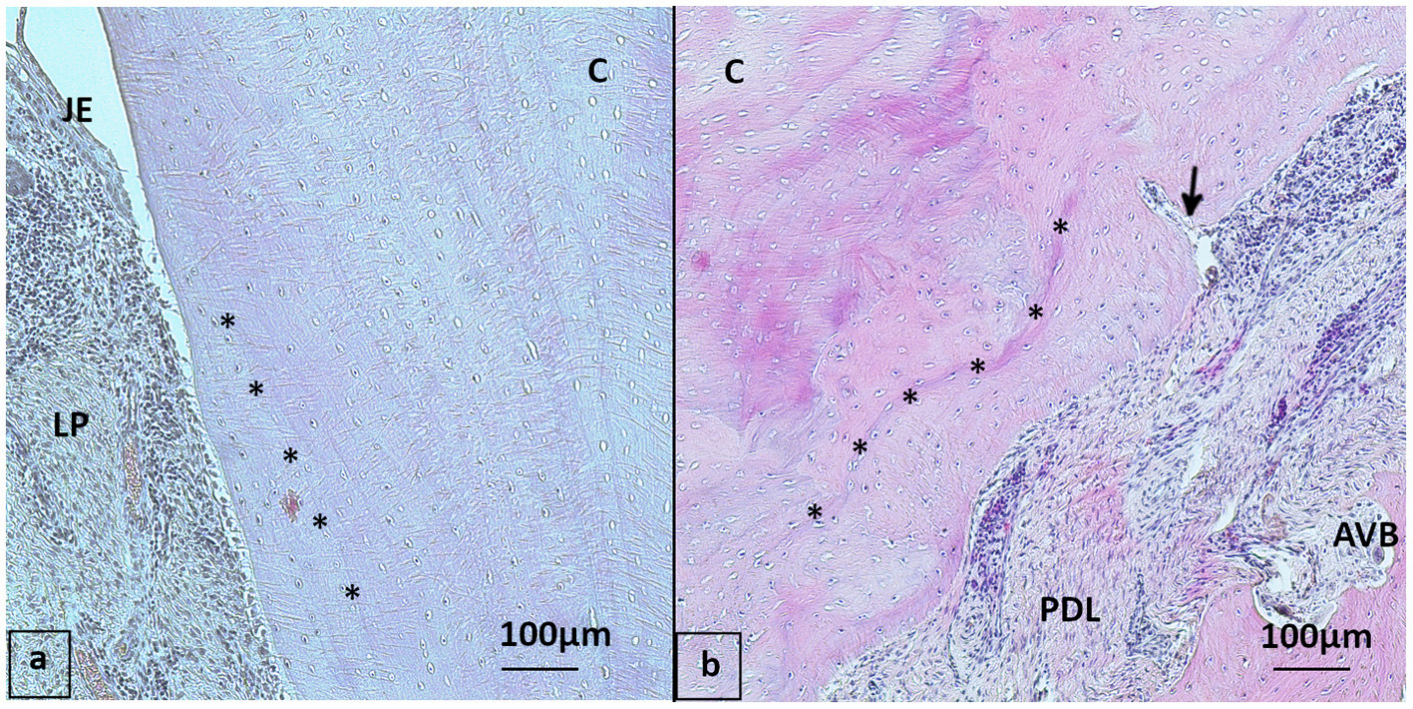
Cemental structure, HE stained **(a)** 20-year-old Icelanic mare from the control group, buccal aspect of 409 with regular cemental structure (asterisk: incremental lines of Salter almost parallel to each other and to the cemental surface). **(b)** 30-year-old Pony gelding from the PPID group, 109 buccal aspect with “irregular” cementum: Multiple signs of reparative cementum and incremental lines of Salter not parallel to each other (asterisk), note the blood vessel (arrow) running into the cementum. JE, Junctional epithelium; LP, Lamina propria; PDL, Periodontal ligament; AVB, Alveolar bone; C, Cementum.

Irregular cemental structures (Figure 7) were found in 15.5% of the PPID group and in 21.3% of the control samples. Within each group, an increase of 1 year in age results in a higher chance for irregular cemental structures (PPID OR: 1.66; control OR: 1.15). The interaction of age and group was significant (*p* = 0.005) in this parameter. Plaque was present within the gingival sulcus in 41.2% of the PPID and in 31.9% of the control samples, but there were no significant differences between the groups or age. Caries was found almost exclusively above the gingival sulcus and was recorded in 48.9% of PPID and 42.6% of control specimens. The interaction between age and group was significant with regard to the presence of caries (*p* = 0.007). In the (older) PPID group, an increase in age by 1 year even yields to a lower chance for caries (OR: 0.75).

### Histological findings in the interdental gingiva

Table 3 shows the detailed results of histological findings in interdental gingiva samples. Age significantly increased (*p* = 0.03) the degree of LI in the PDL. There was no difference between the groups (*p* = 0.91). An increase in age by 1 year yields to a higher chance (OR: 1.38) for high LI in the PDL. Eosinophilic infiltrations in the PDL were increased (*p* = 0.03) in the PPID group with 22.2% (4/18) compared to none in the control group. The interdental cementum of the interdental gingiva in the PPID group showed significantly more irregularities (15/29) than controls (4/31; *p* = 0.02). Comparing the groups mathematically at the same age of 24.4 years, horses affected with PPID had a much higher chance (OR: 8.5) to show irregular cemental structure in interdental positions than controls.

## Discussion

Equine periodontal disease is recognized as a clinically relevant entity, but is often seen as a secondary, mostly reversible process, if macroscopic lesions are corrected. Since the continuous eruption requires ongoing remodeling processes in the equine dentition anyway, healing of periodontal lesions in hypsodont species is assumed to occur with time (9). However, such lesions may lead to quidding, weight loss and early tooth loss when not treated (31, 32). To the knowledge of the authors, this is the first study that describes detailed periodontal, histological findings in PPID affected horses in comparison to unaffected controls. PPID, as an equine specific systemic endocrine disease, impairs multiple metabolic and immunological pathways. However, our hypothesis, that signs of periodontal structural weakening and inflammation in PPID affected horses are more severe than in controls, was supported in only two of our parameters: multifocal to coalescing leukocyte infiltrations in the LP of dental gingiva were found more often in PPID (*p* = 0.002), as well as an irregular cementum in interdental spaces (*p* = 0.02) and at non-interdental sites (age^*^group *p* = 0.005). This might point to a disproportionate inflammation in PPID affected horses and a structural weakening of the periodontal tissue predisposing for the development of diastemata.

### Horses

Five of the ten horses of the PPID group were treated with pergolide. All still expressed classical clinical signs of PPID. A current study from Miller et al. (33) concluded that pergolide does not influence the immune function in horses with PPID. Therefore, we did not expect an influence of pergolide on the parameters we assessed and no separated evaluation of treated and non-treated horses was performed. A limitation of this study might be seen in the significantly older (27.3 ± 2.06 years) PPID than control group (21.4 ± 4.12 years, *p* < 0.001). The inclusion of horses aged at least 15 years could not prevent this effect. In human and canine dentistry, age was shown to be an associated factor for the development of periodontitis (34–37). The term immunosenescence as well as inflammaging (38–40) describes the influence of age on the immune system and getting older not only means a longer exposure time for destructive forces to develop (1, 35, 36). However, our results were statistically controlled for a mean age of 24.4 years and therefore the documented effects of age and PPID are regarded as valid results.

### Histologic findings

#### Gingival epithelia

The predominant presence of a parakeratinized stratified epithelium in the oral gingiva of horses is in line with findings documented for brachydont species like dogs, cats and humans (41–43). The higher amount of orthokeratinized epithelia in controls might reflect a higher mechanical irritation in these sites (8, 44, 45). The types of epithelia within the oral gingiva, sulcular epithelium and junctional epithelium were not significantly different between PPID and controls. Diffusely distributed LI in the gingiva are already documented in a wide range of species and they are claimed to be a normal finding in the gingiva (8, 9, 46, 47). The JE shows unique features compared with other oral epithelia. On one hand, it forms an epithelial barrier against the oral cavity, but permits the passage of extracellular fluid, inflammatory cells and other components of the immunologic host defense system. On the other hand, the function of the JE is to provide a stable connection with the teeth, which is essential for a healthy periodontium (3). Macroscopically horses affected by PPID showed a higher number of gingival sulci of ≥ 1 mm than healthy controls (*p* = 0.004) (28). Our findings here show a higher but not significant number of PPID affected horses (7/97) with a smaller attachment zone or missing contact than controls (4/113). The remodeling and eruption of teeth in hypsodont species requires ongoing cellular renewal and activity in order to keep the JE attached and the gingival sulcus < 1 mm. A gingival sulcus deeper than 1 mm or a loss of attachment could be an expression of a poorer dental status, predisposing for food and bacterial entrapment in the gingival sulcus. In this study we could not show a difference between PPID affected and healthy controls concerning the gingival epithelia.

#### Lamina propria

The collagen fiber arrangement in the LP showed no difference between groups and an irregular fiber network was seen in 7% (8/114) of PPID and 5.2% (6/116) of controls. However, collagen fiber arrangement was evaluated exclusively in HE stained specimens and not in collagen fiber specialized staining methods. Histological evaluations of tendons from PPID affected horses (*n* = 4) showed a reduced longitudinal arrangement of collagen fibers in the suspensory ligament and an accumulation of proteoglycans between suspensory ligament fibers in comparison to two groups of healthy control horses (48). Likewise, in humans with adrenal hypercortisolism, alterations in collagen and epidermal thinning were observed in histological sections from skin with no evidence of leukocyte infiltration (49). In human dentistry, the normal lamina propria is described as collagenous tissue that usually shows no inflammatory infiltrates except in the area of the gingival sulcus (3, 5, 50). The main finding of our study is that PPID affected horses showed significantly (*p* = 0.002) more multifocal to coalescing LI than controls in the LP. Cox et al. (9) described that leukocyte infiltration is a physiological aspect of the equine gingiva mainly in the superficial lamina propria of the free gingiva, while the deep lamina propria contained mild perivascular infiltrations of lymphocytes and plasma cells. In humans, risk factors for severe forms of periodontal disease with LI in LP and even PDL are diseases that influence immune function (e.g., diabetes mellitus, HIV, cushing's syndrome) or lead to exaggerated immune responses (21, 34, 51, 52). In our study, the higher presence of multifocal to coalescing LI in the PPID group could show the onset of immune dysregulation within the LP. The infiltrations consisted mostly of lymphocytes and plasma cells, consistent with a chronic inflammatory/immunological reaction. Interestingly, eosinophils represented the third most common leukocyte cell population within the infiltrates of the lamina propria and in the PDL. The role and function of eosinophils in the periodontium is currently not fully understood. The presence of eosinophils might play a role in gingival protection and might be a transient expression of remodeling rather than a pathological finding (10). Eosinophils beneath the gingival epithelium were reported as a result of parasitic infestation of the gingiva by larvae of botflies (53). In rats with experimentally induced periodontal disease, eosinophils were identified in the lamina propria and are thought to play a role in cell and collagen destruction (9, 54). Our findings of eosinophils are in line with the findings of Cox et al. (9) who found eosinophils “irrespective of the presence or degree of periodontal disease” in the lamina propria and submucosa of their specimens. In contrast, Steinfort et al. (8) found no eosinophils in specimens of horses free from dental and periodontal disorders. Therefore, we assume that the often clustered eosinophils found in this study are a sign of enhanced immunological activity in the gingiva. However, there were no statistical differences detected between groups or related to age in our older probands.

#### Subgingival periodontal ligament

There are few studies in equine dentistry that evaluated the subgingival periodontal ligament and their results are consistent with those of our control group (9, 55). We could not detect a significant difference in the distribution of LI between the groups but 21.2% (24/113) of PPID affected horses showed a multifocal to coalescing distribution of LI in the PDL, while in the control group only 12.4% (14/113) showed this distribution of LI. The reason why only a part of the PPID group in our study showed a higher amount of multifocally to coalescing distributed LI could be the varying amounts of POMC-derived peptides and their multiple systemic effects. PPID is a slowly developing and progressing neurodegenerative disease. Therefore, effects from this disease might be balanced by physiological regulations of the body and its immune system. Recent papers on PPID affected horses showed that they suffer from an impaired immune function (38, 56). Their neutrophil function is decreased resulting in a low oxidative burst capacity and decreased adhesion. Interestingly, an unexpected finding in the study of McFarlane et al. (56) was the increased chemotaxis in healthy aged horses, which is contrary to humans showing an age-related loss of chemotactic ability. On the other hand, age-related inflammation leads to an up-regulation of chemotaxis. In PPID, the age-induced higher LI might be counteracted by immunosuppressant POMC-peptides (e.g., α-MSH and CLIP). This could lead to a multifocal to coalescing high leukocyte infiltration without obvious signs of tissue demarcation, like we have shown in the periodontium.

#### Cementum

On interdental gingiva sites, the PPID group showed more than four times more irregularities in cemental structures than controls (*p* = 0.02). This might still be a normal reaction due to remodeling capacities of the cellular tertiary cementum in hypsodont species (57), possibly caused by trauma or infections, which lead to cemental deposition in resorptive and reparative lesions (2, 58). Irregular cemental structure could also be an age related occurrence (35) as geriatric horses have shorter teeth, which is accompanied by higher tooth mobility. This is counter-regulated by a feature of senile equine dentition: the formation of new cementum replacing and compensating the missing tooth substance (35, 57, 58). However, in our probands, age had no significant influence (*p* = 0.79) on the presence of cemental irregularities. Therefore, we speculate, that PPID might interfere with the building of new cementum.

Nearly all samples in both groups showed plaque within the gingival sulcus or below the gingival epithelium level in interdental locations. In dental locations, caries was found almost exclusively above the gingival sulcus. The nutrition of geriatric horses often involves feed with simpler carbohydrate content (e.g., senior mash with higher starch vs. cellulose content), therefore the high amount of soluble sugars with their cariogenic potential may explain these findings. However, information about diet was not available for the probands. There was no higher prevalence of peripheral caries or plaque in PPID horses. This contradicts the proposal of Simon and Herold (59), who hypothesized that older, but especially PPID affected horses will develop plaque and caries due to immunosuppressive mechanisms.

## Conclusion

Histomorphological characteristics of periodontal tissues of PPID affected horses are described and compared with healthy controls for the first time. This study has some limitations, as the histological evaluations were not made blindly and only hematoxylin-eosin (HE) staining was used. Other stains and techniques for collagen, polarized light and immunohistochemistry for collagen, vascularisation, leukocytes could be further approaches for future studies. PPID affected horses showed a significantly higher prevalence of multifocal to coalescing leukocyte infiltrations within the lamina propria of the gingiva. Further characterization of lymphocytes would be needed in order to evaluate their possible role in destructive periodontal processes in PPID affected horses. However, in the periodontal ligament for the distribution and amount of leukocytic infiltration, age was more important than PPID status.

## Data availability statement

The raw data supporting the conclusions of this article will be made available by the authors, without undue reservation.

## Ethics statement

Ethical review and approval was not required for the animal study because according to German legislation, the post mortal collection of specimens does not need any permission of the animal welfare authority. Written informed consent was obtained from the owners for the participation of their animals in this study.

## Author contributions

AZ wrote the manuscript and performed the histological evaluations of the gingiva. MG performed the histopathological scoring of the pituitary glands. CS and KF contributed to the concept and design of the study. KB performed the statistical analysis. All authors read the manuscript, contributed to manuscript revision, and approved the submitted version.
